# Interactive Effects of Agitation and Cognitive Impairment on Odor Identification in Patients With Late-Life Depression

**DOI:** 10.3389/fpsyt.2022.839012

**Published:** 2022-03-08

**Authors:** Si Zhang, Ben Chen, Xiaomei Zhong, Min Zhang, Qiang Wang, Zhangying Wu, Le Hou, Huarong Zhou, Xinru Chen, Meiling Liu, Mingfeng Yang, Gaohong Lin, Thomas Hummel, Yuping Ning

**Affiliations:** ^1^Department of Geriatric Psychiatry, The Affiliated Brain Hospital of Guangzhou Medical University, Memory Clinic, Guangzhou, China; ^2^Department of Neurology, The Affiliated Brain Hospital of Guangzhou Medical University, Guangzhou, China; ^3^Department of Otorhinolaryngology, Technische Universität Dresden, Smell and Taste Clinic, Dresden, Germany; ^4^The First School of Clinical Medicine, Southern Medical University, Guangzhou, China; ^5^Guangdong Engineering Technology Research Center for Translational Medicine of Mental Disorders, Guangzhou, China

**Keywords:** late-life depression, neuropsychiatric symptoms, agitation, odor identification, cognition, Alzheimer's disease

## Abstract

**Background:**

Late-life depression (LLD) is a risk factor for cognitive decline in older adults, and odor identification (OI) deficits are an early indicator of cognitive decline with LLD. However, neuropsychiatric symptoms (NPSs) are common in LLD and are associated with OI deficits. In subjects with LLD, when OI deficits forecast cognitive decline, whether and how NPS affects the relationship between OI and cognition still must be further explored.

**Objective:**

To comprehensively explore the potential effects of various NPSs on the relationship between OI and cognition in participants with LLD.

**Methods:**

There were 167 patients with LLD and 105 normal elderly (NE) participants. The odor identification test (Sniffin' Sticks), cognitive function assessments (global cognition, memory, executive function, attention, language, visual space), and an NPS assessment (the neuropsychiatric inventory questionnaire) were performed on the subjects. In patients with LLD, the relationship among OI, cognition and NPSs was examined using correlation analysis and moderation analysis.

**Results:**

In patients with LLD, OI was positively correlated with cognition (global cognition, memory, executive function, attention, language) and negatively associated with NPSs (agitation and aberrant motor behavior). In NE group, OI was correlated with executive function. Moderation analysis showed that there was an interactive effect of agitation and cognitive impairment (language deficit or attention deficit) on OI in patients with LLD.

**Conclusion:**

The coexistence of agitation and language or attention deficit was associated with worse OI in subjects with LLD. Agitation should be considered since OI predicts cognitive decline in patients with LLD.

## Introduction

The current prevalence of late-life depression (LLD) is 20% among older adults in China (1). Subjects with LLD have a variety of cognitive deficits, such as deficits memory function, executive function and visuospatial function (2). Previous studies have shown that subjects with LLD were more likely than normal older adults to develop major neurocognitive disorders (3), and a follow-up study suggested that an increased risk of all-cause mortality was affiliated with cognitive impairment and neuropsychiatric symptoms (NPSs) with LLD (4). Therefore, to intervene in a timely manner and enhance quality of life, screening for subjects with LLD who are at high risk for developing dementia in the early stage is essential.

Odor identification (OI) has been regarded as an early marker of preclinical major neurocognitive disorders (5, 6). A review of studies has shown that many brain regions in the olfactory circuit play an important role in cognitive processes, and that damage to these brain regions could lead to simultaneous impairment of cognition and olfactory function (7). The predictive power of OI deficits for developing dementia has been proved in normal elderly individuals (8) and individuals with subjective cognitive decline (SCD) (9), mild cognitive impairment (MCI) (10). Interestingly, our previous studies suggested that OI deficits might also act as a promising predictor of the development of major neurocognitive disorder in subjects with LLD, who with impaired OI not only exhibited poorer cognitive function, but also had more functional and structural impairments in brain regions (11, 12).

However, there is growing evidence showing that olfactory impairment is also associated with other NPSs, which are very common in elderly individuals and might increase the risk of major neurocognitive disorders (13–15). A previous systematic review suggested that 35 to 85% of MCI subjects worldwide have NPSs (16), and the incidence of NPSs is as high as 90% at different stages of AD (17). Moreover, it has been reported that various types of NPSs are associated with olfactory dysfunction, such as agitation, apathy, anxiety and loss of appetite (18, 19). Our previous study suggested that, for elderly individuals, depression moderates the relationship between cognitive impairment and OI (11). In addition, OI dysfunction occurs in many neuropsychiatric diseases, including frontotemporal dementia (20), schizophrenia (21), bipolar disorders (22), autism spectrum disorder (23). Furthermore, behavioral and psychological symptoms in AD, such as agitation, anxiety, and irritability (24), would change upon exposure to fragrance (25).

Overall, there are many joint processing stages among OI dysfunction, cognitive impairment and NPS, and it remains unclear whether and how other NPSs might influence the relationship between OI and cognitive impairment with LLD. Therefore, the aim of this study was to thoroughly explore the potential effects of various NPSs on the relationship between OI and cognition in subjects with LLD. The present results clarify the confounding effects of which NPSs should be considered when applying OI dysfunction to predict the decline of cognitive function with LLD, and to provide a more comprehensive and novel method to understand how OI, cognition and NPS interact with each other.

## Materials and Methods

### Subjects

This study included 167 patients with LLD who came from the Affiliated Brain Hospital of Guangzhou Medical University and 105 normal elderly (NE) adults from the community center. All of the subjects signed informed consent forms after learning about the research content. This study was approved by the ethics committee of the Affiliated Brain Hospital of Guangzhou Medical University. The inclusion criteria for the participants with LLD were as follows: (1) age ≥ 55 years old; (2) meeting the criteria for major depressive disorder in the Diagnostic and Statistical Manual of Mental Disorders, Fourth Edition (DSM-IV); and (3) diagnosed by a clinical psychiatrist. NE participants had normal cognitive function and no history of depression. The exclusion criteria for LLD and NE were as follows: (1) meeting any other diagnostic criteria from the DSM-IV; (2) having hypothyroidism, vitamin B12 deficiency, folic acid deficiency, or syphilis infection; (3) having a history of drug or alcohol abuse; (4) having a neurological disease, such as stroke, white matter injuries, Parkinson's disease, brain tumor, and hydrocephalus; (5) a history of traumatic brain injury; and (6) any condition significantly influencing olfaction, such as sinusitis, nasal polyps, intranasal tumors, congenital or acquired anosmia. All of the subjects were diagnosed by two neuropsychiatrists, one neuropsychologist, and one psychiatrist.

### Neuropsychological Assessments

The neuropsychological assessments included the following: (1) global cognition: Mini-Mental State Examination (MMSE); (2) memory: Auditory Verbal Learning Test (AVLT) (Sum of N1, N2, N3, N4 and N5 scores); (3) executive function: Trail Making Test (TMT) A and B; (4) attention: Digital Span Test (DST); (5) language ability: animal Verbal Fluency Test (VFT); and (6) visual space skill: Clock Drawing Test (CDT). Because the TMT-A and TMT-B are tested under a time limit, less time shows better capability, and assessing this capability uses the inverse of the scores. NPSs were evaluated using the neuropsychiatric inventory questionnaire (NPI). The total score of the NPI is the sum of the scores for 12 items: delusions, hallucinations, agitation, dysphoria, anxiety, euphoria, apathy, disinhibition, irritability, aberrant motor behavior, nighttime behavioral disturbances, and appetite. The score for each symptom is the product of frequency and severity. Depressive symptoms were assessed by the Hamilton Depression Rating Scale-17 (HAMD-17).

### Olfactory Assessments

The Sniffin' Sticks Screen 16 test evaluates OI (26), and 16 pens had different odors. Subjects smelled one pen, they smelled the next pen after 20 s, and they smelled each pen ~3–4 s. After smelling a pen, the subjects were given four options to define the odor they had just smelled. To avoid the effects of eating on olfactory function, subjects were forbidden to eat any food (including water) for 30 min. The subjects completed an investigation of whether there was a history of impairment of olfactory function. One the day of the neuropsychological assessments, the subjects were given the OI test.

### Statistical Analyses

SPSS software, version 25.0 (IBM, Chicago, Illinois, USA) was used for all analyses, and statistical significance was defined as *p* < 0.05. Continuous variables are represented as the means ± standard deviations, and categorical variables are represented as percentages and numbers. One-way analysis of variance (ANOVA) was used to compare demographic and clinical variables between the LLD and NE groups for the continuous variables. The chi-square (χ^2^) test was used to compare demographic and clinical variables between the LLD and NE groups for the categorical variables. Partial correlations were used to analyze the associations among OI, cognitive scores and NPI scores, adjusted for sex, and years of education.

To detect the interaction effect, PROCESS software (27) was used to analyze regressions. The model entered predictor variables (each item's cognitive score) and the interaction term (each item's NPI score) at the same time. OI served as an outcome variable, and age was a control variable in the model. Regression equations were constructed to perform simple slope analysis. The relationship between the predictor variable and the outcome variable was explored at high (+1 SD) and low (−1 SD) levels of the interacting variable. Confidence intervals determined the significance of the results explored by PROCESS software. Zero was not in the significant interval.

## Results

### Demographic Data

The demographic data of the NE and LLD groups are listed in Table 1. There were no significant differences in age, smoking history, hypertension history, or diabetes history between the NE and LLD groups (*p* > 0.05). The NE group had a significantly lower percentage of women, more years of education, higher OI scores and cognitive scores and lower NPI scores than the LLD group (*p* < 0.05) (see Table 1).

**Table 1:** Demographic data, clinical information, cognitive and olfactory function.

	**NE (*n* = 105)**	**LLD (=167)**	**F/** **χ^2^**	***p***
Female gender	65 (61.9%)	134 (80.2%)	11.037	0.001^*^
Age	67.3 ± 6.5	67.4 ± 7.2	−0.006	0.996
Years of education	11.3 ± 3.5	8.8 ± 3.7	5.528	<0.001^*^
OI	11.1 ± 2.3	9.8 ± 2.8	3.708	<0.001^*^
Former smoker	19 (18.1%)	19 (11.4%)	2.421	0.120
Hypertension	37 (35.2%)	53 (31.7%)	0.357	0.550
Diabetes	13 (15.1%)	23 (13.8%)	0.109	0.742
HAMD	2.3 ± 3.0	8.8 ± 7.1	−8.975	<0.001^*^
Early-onset/Late-onset	–	36/131	–	–
Onset of depression	–	79 (47.3%)	–	–
Cognitive function				
MMSE	26.9 ± 2.6	22.8 ± 5.3	7.160	<0.001^*^
AVLT N1-N5 total	28.8 ± 12.3	17.0 ± 5.9	6.028	<0.001^*^
TMTA-Time	47.2 ± 15.8	67.2 ± 30.5	−6.170	<0.001^*^
TMTB-Time	59.8 ± 23.1	87.5 ± 37.8	−6.638	<0.001^*^
VFT	15.3 ± 4.1	12.7 ± 3.9	4.935	<0.001^*^
CDT	3.9 ± 0.4	3.3 ± 0.8	6.202	<0.001^*^
DST	10.1 ± 2.0	9.0 ± 2.4	3.795	<0.001^*^
Neuropsychiatric symptoms
NPI total	3.8 ± 6.6	17.6 ±16.0	−8.391	<0.001^*^
Delusions	0	0.3 ± 1.4	−2.319	0.021^*^
Hallucinations	0	0.2 ± 1.1	−1.885	0.061
Agitation	0.3 ± 1.1	1.4 ± 2.5	−4.098	<0.001^*^
Dysphoria	0.4 ± 1.6	3.8 ± 4.2	−8.136	<0.001^*^
Anxiety	0.8 ± 2.1	3.9 ± 3.9	−7.518	<0.001^*^
Euphoria	0	0 ± 0.2	−0.792	0.429
Apathy	0.2 ± 0.8	1.4 ± 3.0	−3.979	<0.001^*^
Disinhibition	0	0.2 ± 1.2	−1.408	0.160
Irritability	0.5 ± 1.3	1.1 ± 2.3	−2.763	0.006^*^
Aberrant Motor Behavior	0	0.4 ± 1.6	−2.150	0.032^*^
Nighttime Behavioral Disturbances	1.6 ± 3.0	4.0 ± 4.2	−5.044	<0.001^*^
Appetite	0.1 ± 0.6	1.1 ± 2.8	−3.449	0.001^*^

*Age, sex, and year of education were included as covariates when comparing the cognitive scores and OI between the NE group and LLD group. NE, normal elderly; LLD, late-life depression; OI, odor identification; HAMD, Hamilton Depression Rating Scale; MMSE, Mini-Mental State Examination; AVLT, Auditory Verbal Learning; TMT, Trail-Making Test; VFT, Verbal Fluency Test; CDT, Clock Drawing Test; DST, Digital Span Test; NPI, neuropsychiatric inventory questionnaire*;

* *p < 0.05*.

### Correlation Analyses

**Figure 1:**
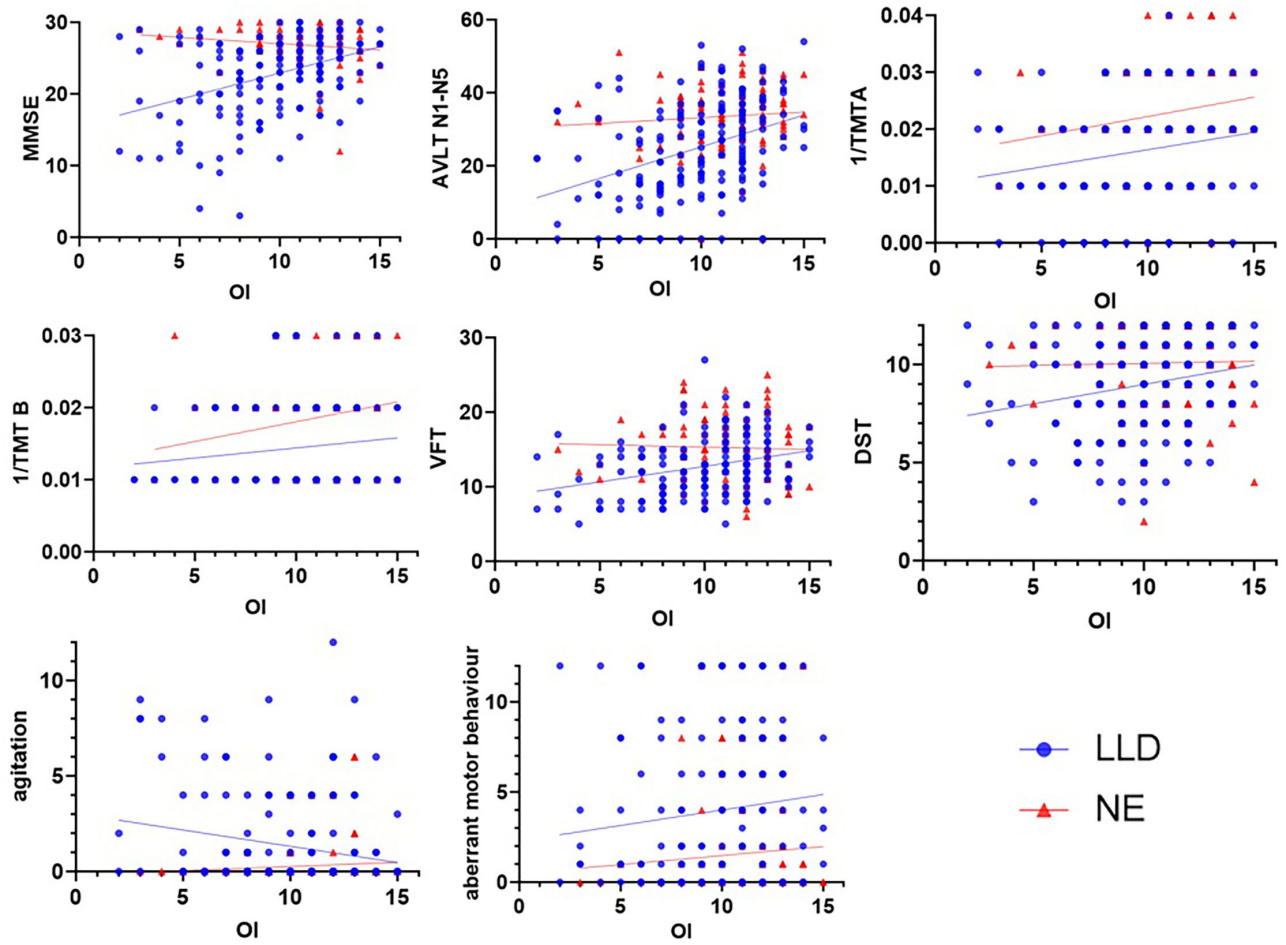
Correlations among OI, cognitive scores and NPI scores. In the NE group, OI was correlated with 1/TMTA (*r* = 0.231, *p* = 0.020) and 1/TMTB (*r* = 0.212, *p* = 0.034). In the LLD group, OI was correlated with MMSE (*r* = 0.369, *p* < 0.001), AVLT N1-N5 total (*r* = 0.351, *p* < 0.001), 1/TMTA (*r* = 0.184, *p* = 0.025), 1/TMTB (*r* = 0.195, *p* = 0.019), VFT (*r* = 0.288, *p* < 0.001), and DST (*r* = 0.220, *p* = 0.007); OI was correlated with NPI scores, including agitation (*r* = −0.200, *p* = 0.010) and aberrant motor behavior (*r* = −0.165, *p* = 0.035). LLD, late-life depression; NE, normal elderly; MMSE, Mini-Mental State Examination; AVLT, Auditory Verbal Learning; TMT, Trail-Making Test; VFT, Verbal Fluency Test; DST, Digital Span Test.

OI was associated (all *p* values < 0.05) with all cognitive scores in all subjects, including MMSE (*r* = 0.31), AVLT N1-N5 total (*r* = 0.32), 1/TMTA-Time (*r* = 0.25), 1/TMTB-Time (*r* = 0.25), VFT (*r* = 0.21), CDT (*r* = 0.14), and DST (*r* = 0.18). Furthermore, OI was correlated with NPI scores, including agitation (*r* = −0.18), dysphoria (*r* = −0.14), anxiety (*r* = −0.12), apathy (*r* = −0.14), and aberrant motor behavior (*r* = −0.16). In the NE group, OI was correlated with 1/TMTA-Time (*r* = 0.23) and 1/TMTB-Time (*r* = 0.21). In the LLD group, OI was correlated with MMSE (*r* = 0.37), AVLT N1-N5 total (*r* = 0.35), 1/TMTA-Time (*r* = 0.18), 1/TMTB-Time (*r* = 0.20), VFT (*r* = 0.29), and DST (*r* = 0.22); OI was associated with NPI scores, including agitation (*r* = −0.20) and aberrant motor behavior (*r* = −0.17) (see Table 2; Figure 1).

**Table 2:** Correlations between OI and cognition, OI and NPI.

	**All subjects**		**NE**		**LLD**	
	***r***	***p***	***r***	***p***	***r***	***p***
MMSE	0.311	<0.001^*^	−0.142	0.155	0.369	<0.001^*^
AVLT N1-N5 total	0.316	<0.001^*^	0.107	0.280	0.351	<0.001^*^
1/TMTA-Time	0.246	<0.001^*^	0.231	0.020^*^	0.184	0.025^*^
1/TMTB-Time	0.245	<0.001^*^	0.212	0.034^*^	0.195	0.019^*^
VFT	0.205	0.001^*^	−0.025	0.803	0.288	<0.001^*^
CDT	0.136	0.031^*^	0.030	0.764	0.125	0.129
DST	0.181	0.001^*^	0.051	0.615	0.220	0.007^*^
Delusions	−0.056	0.358	-	-	−0.031	0.695
Hallucinations	−0.089	0.147	-	-	−0.084	0.289
Agitation	−0.182	0.003^*^	-	-	−0.200	0.010^*^
Dysphoria	−0.144	0.018^*^	-	-	−0.076	0.333
Anxiety	−0.121	0.047^*^	-	-	−0.067	0.393
Euphoria	0.060	0.325	-	-	0.097	0.220
Apathy	−0.142	0.020^*^	-	-	−0.132	0.093
Disinhibition	0.011	0.860	-	-	0.063	0.428
Irritability	−0.024	0.695	-	-	−0.001	0.989
Aberrant motor behavior	−0.162	0.008^*^	-	-	−0.165	0.035^*^
Nighttime behavioral disturbances	0.071	0.249	-	-	0.146	0.064
Appetite	−0.063	0.307	-	-	−0.038	0.633
NPI	−0.133	0.029^*^	-	-	−0.079	0.317

*NE, normal elderly; LLD, late-life depression; OI, odor identification; MMSE, Mini-Mental State Examination; AVLT, Auditory Verbal Learning; TMT, Trail-Making Test; VFT, Verbal Fluency Test; CDT, Clock Drawing Test; DST, Digital Span Test; NPI, neuropsychiatric inventory questionnaire*;

* *p < 0.05*.

### Testing the Hypothesized Interaction

Considering the significant associations between OI and two NPI symptoms (agitation and aberrant motor behavior) in LLD, we further analyzed the interactive effects of these two NPI items and cognitive impairment on OI. In the NE group, OI was not related to NPI symptoms, so the interaction analysis could not be performed.

**Figure 2:**
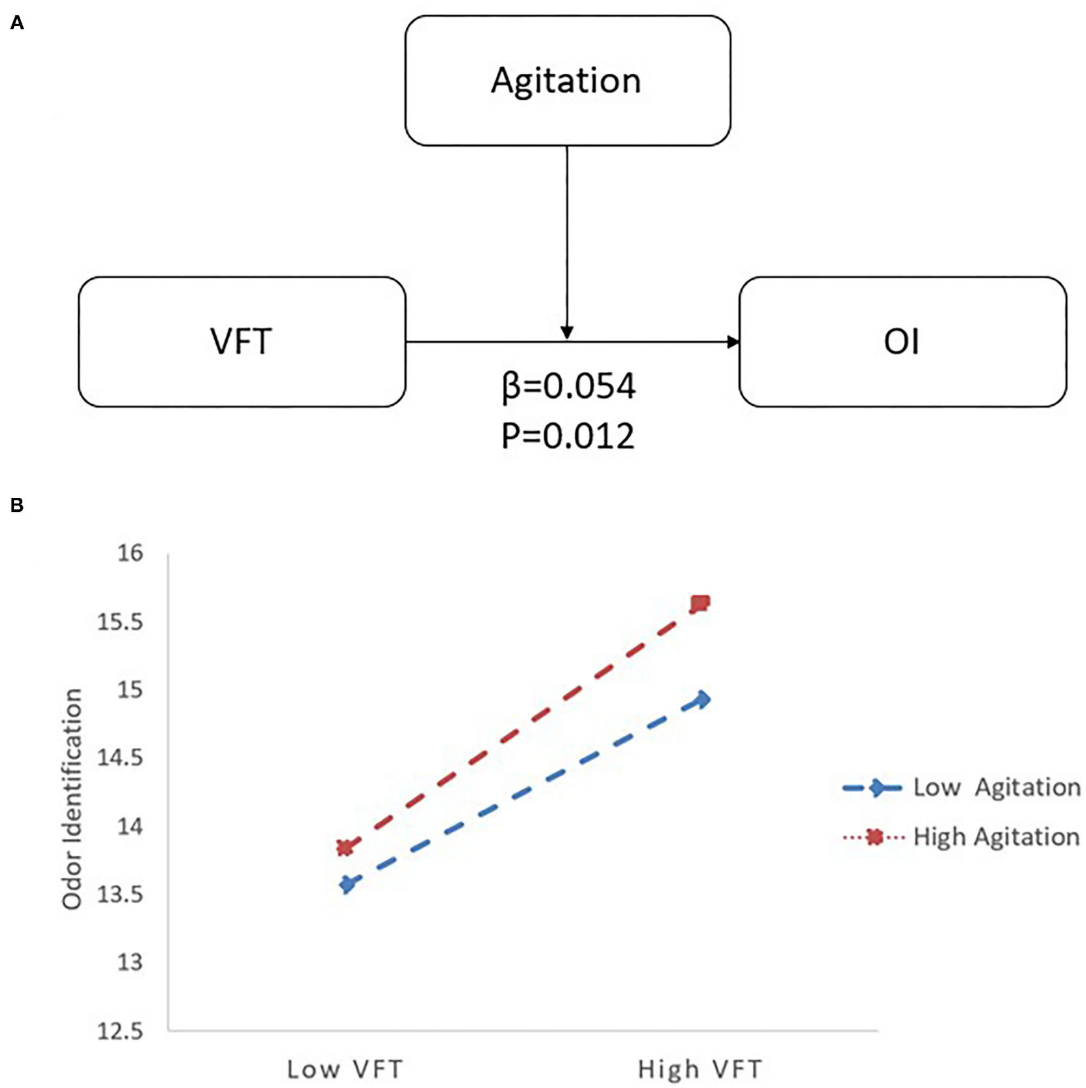
The moderating effect of agitation and VFT on OI with LLD. **(A)** The interactive model among agitation, VFT and OI. There is an interactive effect of VFT*Agitation (β = 0.054, *p* = 0.012) on OI. **(B)** When agitation increased from low (−1 SD; *B* = 0.108) to high (+1 SD; *B* = 0.305) values, the positive association between VFT and OI became significantly stronger by follow-up simple slope analysis. LLD, late-life depression; VFT, Verbal Fluency Test; OI, odor identification.

In the LLD group, when VFT served as a predictor variable, OI was significantly correlated with age (*B* = −0.091, 95% CI [−0.150, −0.032], *p* < 0.05), VFT (*B* = 0.177, 95% CI [0.069, 0.285], *p* < 0.05), and agitation (*B* = −0.210, 95% CI [−0.386, −0.035], *p* < 0.05). Additionally, there was an interaction between VFT and agitation (*B* = 0.054, 95% CI [0.012, 0.096], *p* < 0.05) on OI (*R*^2^ = 0.197; *p* < 0.05) (see Table 3). Follow-up simple slope analysis revealed that the positive association between VFT and OI became significantly stronger as agitation increased from low (−1 SD; *B* = 0.108, 95% CI [−0.014, 0.229], *p* > 0.05) to high (+1 SD; *B* = 0.305, 95% CI [0.159, 0.450], *p* < 0.05) values (see Figure 2).

**Table 3:** Multiple linear regression analysis of the potential impact factors on OI with LLD.

**Variable**	**β**	**SE**	***t***	***p***	**95%CI**
**Model 1**
Age	−0.091	0.030	−3.058	0.003	(−0.150, −0.032)
VFT	0.177	0.055	3.240	0.002	(0.069, 0.285)
Agitation	−0.210	0.089	−2.371	0.019	(−0.386, −0.035)
VFT*Agitation	0.054	0.021	2.552	0.012	(0.012, 0.096)
**Model 2**
Age	−0.092	0.029	−3.121	0.002	(−0.149, −0.034)
DST	0.242	0.089	2.730	0.007	(0.067, 0.417)
Agitation	−0.193	0.092	−2.097	0.038	(−0.374, −0.011)
DST*Agitation	0.085	0.038	2.234	0.027	(0.010, 0.160)

*VFT, Verbal Fluency Test; DST, Digital Span Test*.

**Figure 3:**
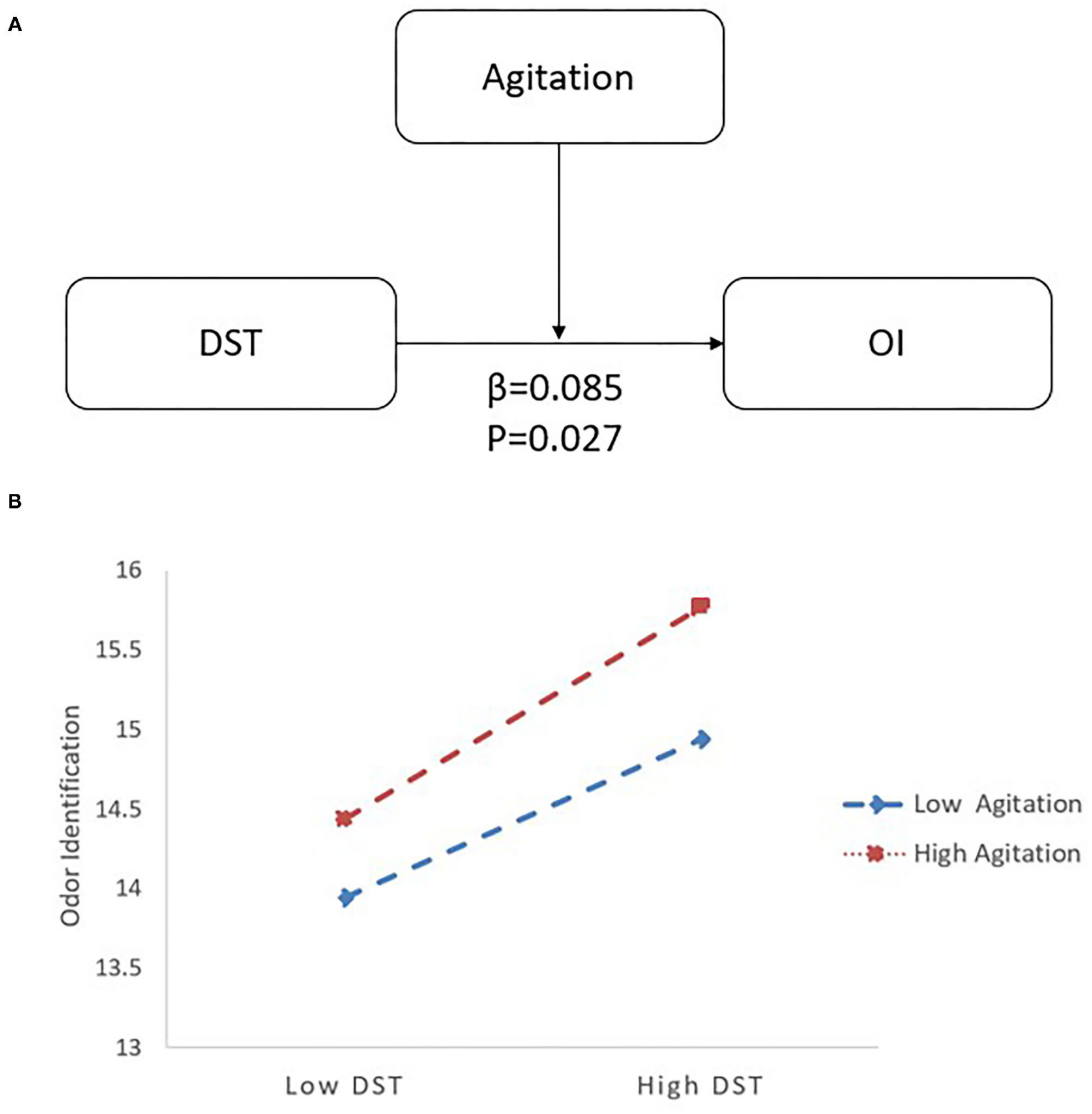
The interactive effect of agitation and DST on OI with LLD. **(A)** The interactive model among agitation, DST and OI. There is an interactive effect of DST*Agitation (β = 0.085, *p* = 0.027) on OI. **(B)** When agitation increased from low (−1 SD; *B* = 0.134) to high (+1 SD; *B* = 0.440) values, the positive association between DST and OI became significantly stronger by follow-up simple slopes analysis. DST, Digital Span Test; OI, odor identification; LLD, late-life depression.

In the LLD group, when DST served as a predictor variable, OI was significantly correlated with age (*B* = −0.092, 95% CI [−0.149, −0.034], *p* < 0.05), DST (*B* = 0.242, 95% CI [0.067, 0.417], *p* < 0.05), and agitation (*B* = −0.193, 95% CI [−0.374, −0.011], *p* < 0.05). Additionally, there was an interaction between DST and agitation (*B* = 0.085, 95% CI [0.010, 0.160], *p* < 0.05) on OI (*R*^2^ = 0.168; *p* < 0.05) (see Table 3). Follow-up simple slopes analysis revealed that the positive association between DST and OI became significantly stronger as agitation increased from low (−1 SD; *B* = 0.134, 95% CI [−0.059, 0.327], *p* > 0.05) to high (+1 SD; *B* = 0.440, 95% CI [0.183, 0.696], *p* < 0.05) values (see Figure 3).

When other cognitive scores (MMSE, AVLT N1-N5, 1/TMTA-Time, 1/TMTB-Time) were used as predictor variables in the LLD group, there were no significant correlations between cognitive scores and agitation, and the interaction terms between cognitive scores and each of agitation symptoms were not associated with OI, including MMSE^*^agitation (*p* = 0.113), AVLT N1-N5^*^agitation (*p* = 0.584), 1/TMTA-Time ^*^agitation (*p* = 0.222), 1/TMTB-Time (*p* = 0.789) and 1/TMTB-Time ^*^agitation (*p* = 0.006). Furthermore, no significant interactive effect was found in aberrant motor behavior regarding the associations between OI deficits and cognitive impairment.

## Discussion

The present study comprehensively analyzed the interactive effects of NPSs and cognitive impairment on OI in patients with LLD and mainly found three results. First, in different cognitive domains, global cognition, memory, executive function, language, and attention performance were positively associated with OI scores in patients with LLD. Second, OI scores were negatively correlated with NPI scores, including agitation and aberrant motor behavior in patients with LLD. Third, the complication by agitation of cognitive impairment (language or attention deficit) would lead to worse OI, suggesting a relationship between OI and cognitive function changes as agitation increases or decreases in patients with LLD.

Consistent with our previous research in elderly individuals (11, 12, 28), there is a positive correlation between OI and cognitive performance. OI deficits could serve as a marker of cognitive impairment (8, 10) because the olfactory pathway and cognitive circuit share many brain areas (7). Murphy provided a hypothesis about how olfactory deficits interact with cognitive impairment: if a patient with olfactory dysfunction wants to remember or identify an odor, the olfactory and brain areas with cognitive function will be hyperactive. In one's life, neurodegeneration leads to structural abnormalities of the brain with cognitive impairment and accelerates the transition to AD (29). For patients with LLD, our previous study suggested that OI deficits could serve as a promising predictor of developing major neurocognitive disorder, and neuroimaging showed that OI deficits and cognitive function had additive effects on the super synchronization of the hippocampus or fusiform gyrus (12). However, whether NPSs, which are very common in patients with LLD, might influence the relationship between OI deficits and cognitive impairment remains unknown.

The current study suggested that OI scores were negatively correlated with NPSs in patients with LLD, including agitation, apathy and aberrant motor behavior. In addition, agitation and cognitive impairment (language deficit or attention deficit) showed an interactive effect on OI in NPSs, consistent with previous research that olfactory dysfunction, cognitive impairment and agitation are strongly associated with each other. Regarding to the relationships between agitation and OI, previous studies have indicated that numerous brain regions related to agitation also participate in the olfactory circuit, such as the hippocampus, amygdala, insula, orbitofrontal cortex and habenula nucleus (30, 31). Most studies found that the application of olfactory stimulation using essential oils, such as lavender or lemon, could improve the symptoms of agitation (32–34). With respect to the relationships between agitation and cognitive function, previous studies have shown that language, attention and other aspects of cognitive function are improved when agitation symptoms are alleviated (35, 36). Additionally, agitation was associated with structural and functional abnormalities of specific areas of the gray and white matter in subjects with cognitive impairment (37). The structure of the olfactory network and limbic system in brain regions could shed light on the relationship among agitation, OI and cognitive function and could explained their deficits in conjunction with different neuropsychiatric diseases. A large number of these regions have been regarded as providing the neural basis for neuropsychiatric symptoms, olfactory processing and cognitive processing, including the amygdala, insula, hippocampus, orbitofrontal cortex and anterior cingulate cortex. Thus, the present results are an essential supplement to our previous study showing that there is a moderating effect between depression and cognitive impairment (global cognition, memory and language) on OI (28). This finding suggests that we should comprehensively evaluate agitation and exclude its confounding effects when applying OI to predict cognitive decline (language and attention) in patients with LLD.

Apart from agitation, the present study also suggested that aberrant motor behavior had a significant effect on OI. In line with a previous study showing that OI dysfunction was connected to frontotemporal dementia with changes in behavior and personality (38), our results suggested that OI was associated with aberrant motor behavior in patients with LLD. The mechanism remains unclear and must be further explored. Overall, although aberrant motor behavior was associated with OI in patients with LLD, it did not exhibit an interactive effect, such as with agitation. Therefore, it is not necessary to exclude the possible confounding effects of aberrant motor behavior when OI predicts cognitive decline in patients with LLD.

In this study, years of education was not set as a moderator because most of the studies supported that education was not a factor affecting OI. According to a review study by James, the relationship between education level and olfactory function is currently debated and must be further explored (39). Some studies have shown that there is no relationship between OI and years of education (40). But the study has shown that economic status and educational level were significantly related to OI, reflecting that the degree of exposure to foreign substances leads to differences in familiarity with smells or their names (41). Simplifying testing or using a more world-wide approach to testing is the solution to this problem (42). Overall, the relationship between OI and education level needs to be explored comprehensively in future study.

There are several limitations to our study. First, the current study is a cross-sectional study; whether alterations in NPS may affect OI performance and whether NPS have a mixed effect when using OI predict cognitive decline in patients with LLD needs to be further demonstrated in follow-up studies. Second, the current study only analyzed behavior, but the underlying mechanism of the mediating effect of NPS on cognition and OI is still unclear. We look forwards to future neuroimaging studies to find the mechanism to better understand their relationship. Third, the current study did not rule out the effects of drugs that patients with LLD took many different antidepressants. Fourth, although patients with Parkinson's disease was excluded, depression and olfactory dysfunction can be early symptoms of Parkinson's disease without motor symptoms, which could have been a potential confounding factor in the present study.

Overall, the coexistence of agitation and language or attention deficit was associated with worse OI in patients with LLD, and research exploring the relationship between OI and cognitive function should consider as an analysis of agitation and regulate its mixed effects. The present results provide a multilevel and comprehensive understanding of how NPS, cognition and OI influence each other, and they could be helpful for the realistic use of OI in clinical practice.

## Data Availability Statement

The raw data supporting the conclusions of this article will be made available by the authors, without undue reservation.

## Ethics Statement

The studies involving human participants were reviewed and approved by the Ethics Committees of the Affiliated Brain Hospital of Guangzhou Medical University. The patients/participants provided their written informed consent to participate in this study.

## Author Contributions

All authors contributed to the writing and revision of the manuscript. All authors have read and approved of the final manuscript.

## Funding

This study was supported by a grant from the National Natural Science Foundation of China (Nos. 81701341, 82101508, and 82171533), Guangzhou Municipal Psychiatric Diseases Clinical Transformation Laboratory (No: 201805010009), Key Laboratory for Innovation Platform Plan, the Science and Technology Program of Guangzhou, China, Science and Technology Plan Project of Guangdong Province (No. 2019B030316001), and National Key Research and Development Program of China (No. 2016YFC0906300).

## Conflict of Interest

The authors declare that the research was conducted in the absence of any commercial or financial relationships that could be construed as a potential conflict of interest.

## Publisher's Note

All claims expressed in this article are solely those of the authors and do not necessarily represent those of their affiliated organizations, or those of the publisher, the editors and the reviewers. Any product that may be evaluated in this article, or claim that may be made by its manufacturer, is not guaranteed or endorsed by the publisher.
